# Time Does Not Heal All Ills: The Late Occurrence of Hemolytic Anemia After Prosthetic Mitral Valve Replacement

**DOI:** 10.7759/cureus.26348

**Published:** 2022-06-26

**Authors:** Preetraj Kaur, Huda Fatima

**Affiliations:** 1 Internal Medicine, Mercy Health — St. Vincent Medical Center, Toledo, USA

**Keywords:** anemia and hyperbilirubinemia, mechanical shear forces, hemolytic anemia, prosthetic valves, hemolysis

## Abstract

Hemolytic anemia is a well-known complication of prostheses following the replacement of cardiac valves. Therefore, in all patients with prosthetic valve placement who present with anemia or hyperbilirubinemia, hemolysis is an important differential that must be ruled out, irrespective of how long the valve replacement has lasted. Indications for repair either through percutaneous or surgical approach generally include the severity of hemolysis based on either persistent symptoms of fatigue, the continued requirement of blood transfusions, or else the progression of valvular dysfunction. However, if hemoglobin remains stable, symptoms resolve, there is minimal functional impairment, or the risk of comorbidities is greater than the benefits of invasive intervention, then an initial trial of medical therapy is prudent. Our case report is unique as it demonstrates the late occurrence of symptomatic severe hemolytic anemia more than 20 years after valvular replacement. It also offers an alternative approach to invasive management in patients who develop this complication after such a prolonged asymptomatic period.

## Introduction

Hemolytic anemia is a well-known complication of prostheses following the replacement of cardiac valves, reported in approximately 15% of cases [1]. Bio-prosthetic valves have distinct advantages over mechanical valves including decreased risk of thromboembolism, non-requirement of oral anticoagulants, and a hemodynamically favorable central flow orifice with a lower risk of hemolytic anemia [2]. Literature would indicate that hemolytic anemia is no longer a complication with porcine prostheses in the absence of a paravalvular leak [3]. In comparison, mechanical valves are more prone to causing hemolysis with aortic mechanical valves reported to have a higher percentage of hemolytic anemia than mitral valves [4]. Our case report is unique as it demonstrates the late occurrence of symptomatic severe hemolytic anemia more than 20 years after valvular replacement in a patient who initially had a porcine bio-prosthetic mitral valve placed in 1992 followed by mechanical mitral valve placement in 1999. Moreover, it offers an alternate approach to management in patients who develop this complication after such a prolonged asymptomatic period.

## Case presentation

An 86-year-old African American female was sent to our tertiary care medical center's ER by her primary care physician due to a chief presenting complaint of one episode of dark-colored stool along with a lab result of hemoglobin 5.5 g/dL. She also gave a history of symptoms of fatigue, generalized weakness, and dyspnea on exertion, which had been ongoing for the last several weeks. Her past medical history was significant for peptic ulcer disease, gastritis, duodenitis, hypertension, hyperlipidemia, chronic obstructive pulmonary disease (COPD), bioprosthetic (pig) mitral valve replacement in 1992 followed by mechanical mitral valve replacement in 1999, chronic atrial fibrillation on warfarin therapy, and congestive heart failure with preserved ejection fraction. The patient had recently undergone an esophagogastroduodenoscopy (EGD) two months before admission for symptoms of persistent gastritis, which revealed a gastric ulcer measuring 15 mm in diameter. A biopsy had been taken during the EGD which came back positive for *Helicobacter pylori* and the patient had been successfully treated. On the day of admission, the patient was evaluated and found to be hemodynamically stable and afebrile. Her cardiovascular examination revealed irregularly irregular rhythm, loud S1, and a pan systolic murmur located at the apex and radiating towards the axilla. The patient’s lung examination showed bilaterally clear breath sounds without any rales or crackles. Abdominal examination showed a soft, non-tender, and non-distended abdomen. No lower extremity edema was found bilaterally. Her social history was insignificant and her family history was unremarkable for any bleeding disorders. Her home medications included warfarin for chronic atrial fibrillation.

The patient’s lab work revealed macrocytic anemia with a hemoglobin of 5.6 g/dL, hematocrit of 19.5 %, mean corpuscular volume (MCV) of 101 fL, red cell distribution width of 22.5 %, a platelet count of 253 x 1000 µ/L, a WBC count of 6.0 x 1000 µ/L, and an international normalized ratio (INR) of 1.1, as shown in Table 1. Her serum creatinine and serum blood urea nitrogen (BUN) remained stable as shown in Table 2. Peripheral blood smear showed normocytic anemia with anisocytosis, polychromasia, and the presence of schistocytes. The other two cell lines had normal morphology on peripheral blood smear pathology. Further laboratory studies showed lactate dehydrogenase (LDH) 1278 U/l, a reticulocyte count of 7.6%, and a serum haptoglobin of <10 g/dL as shown in Table 3. Iron studies, serum folate, and B12 were unremarkable (Table 3). However, liver function tests revealed increased bilirubin levels of 5.58 mg/dL with indirect hyper-bilirubinemia of 5.14 mg/dL (Table 4). Further workup including cardiac enzymes and a chest x-ray was normal. The patient was transfused with two units of packed red blood cells on the day of admission, thereby improving hemoglobin to 7.7 g/dL the following morning (Table 5). Gastroenterology was consulted and the patient was scheduled for a repeat EGD to rule out peptic ulcer disease, which revealed previous ulcers had completely healed and no evidence or stigmata of bleeding. The patient also underwent a colonoscopy to rule out lower gastrointestinal (GI) causes of blood loss. Colonoscopy revealed no evidence of bleeding, mucosal abnormalities, or any stigmata of bleeding. 

**Table 1:** Complete blood count, partial thromboplastin time, and the international normalized ratio WBC: White blood cell, RBC: Red blood cell, MCV: Mean corpuscular volume, MCH: Mean corpuscular hemoglobin, MCHC: Mean corpuscular hemoglobin concentration, RDW: Red cell distribution width, MPV: Mean platelet volume, PTT: Partial thromboplastin time, INR: International normalized ratio, DAT: Direct antibody test

WBC (3.5 - 11.3 K/u/L)	6.0
RBC (3.95 - 5.11 m/uL)	1.93
Hemoglobin (11.9 - 15.1 g/dL)	5.6
Hematocrit (36.3 - 47.1 %)	19.5
MCV (82.5 - 102.9 fL)	101.0
MCH (25.2 - 33.5 PG)	29.0
MCHC (28.4 - 34.8 g/dL)	28.7
RDW (11.8 - 14.4%)	22.5
Platelets (138 - 453 k/uL)	253
MPV (8.1 - 13.5 fL)	10.3
Morphology	Anisocytosis present
Morphology	1+ Polychromasia
Morphology	1+ Schistocytes
PTT (9.1 - 12.3 sec)	11.7
INR	1.1
DAT, Polyspecific	Negative

**Table 2:** Basic metabolic panel CO2: Bicarbonate, BUN: Blood urea nitrogen, GFR: Glomerular filtration rate

Sodium (135 – 145 mmol/L)	136
Potassium (3.7 - 5.3 mmol/L0)	4.6
Chloride (98 - 107 mmol/L)	105
CO2 (20 - 31 mmol/L)	18
BUN (8 - 23 mg/dL)	33
Creatinine (0.50 - 0.90 mg/dL) icteric specimen	0.62
Anion Gap (9 - 17 mmol/L)	13
GFR African American ( > 60 mL/min)	>60
Glucose (70 - 99 mg/dL)	103
Calcium (8.6 - 10.4 mg/dL)	8.9

**Table 3:** Further workup for anemia TIBC: Total iron-binding capacity

Reticulocyte % (0.5 - 1.9%]	7.6
Absolute Reticulocyte Number (0.030- 0.080 M/uL)	0.190
Immature Reticulocyte Number (2.7 - 18.3%)	32,600
Reticulocyte Hemoglobin (28.2 - 35.7 pg)	25.5
Haptoglobin (30 - 200 mg/dL)	<10
Hemoglobin, Free, Plasma (0.0 - 9.7 mg/dL)	86.0
Lactate Dehydrogenase (135 - 214 U/L)	1278
Iron (37 - 145 ug/dL)	84
TIBC (250 - 450 ug/dL)	313
Iron Saturation (20 - 55%)	27
Vitamin B-12 (232 - 1245 pg/mL)	419
Folate (> 4.8 ng/mL)	15.3

**Table 4:** Liver function tests ALT: Alanine transaminase, AST: Aspartate aminotransferase

Albumin (3.5 - 5.2 g/dL]	3.8
Alkaline Phosphatase (35 - 104 U/L)	88
ALT (5 - 33 U/L)	14
AST ( < 32 U/L)	51
Total Bilirubin (0.3 - 1.2 mg/dL)	5.58
Bilirubin, Direct ( < 0.31 mg/dL)	0.44
Bilirubin, Indirect (0.00 - 1.00 mg/dL)	5.14
Total protein (6.4 - 8.3 g/dL)	6.1
Albumin/Globulin Ratio ([1.0 - 2.5)	1.7

**Table 5:** Hemoglobin trend during hospitalization

Day 1	5.5 g/dL
Day 2	7.7 g/dL
Day 3	8.3 g/dL
Day 4	6.4 g/dL
Day 5	8.6 g/dL
Day 6	9.8 g/dL
Day 7	9.3 g/dL
Day 8	9.1 g/dL
Day 9	9.2 g/dL

With both upper and lower GI bleed ruled out and laboratory studies suggestive of hemolytic anemia, hematology-oncology was consulted for further workup. A Coombs test was completed which was negative. Ultrasounds of the spleen and liver were unremarkable for hepato-splenomegaly. Serum protein electrophoresis showed normal results (Table 6).

**Table 6:** Hemoglobin electrophoresis Total prot sum: Total sum of individual serum proteins, Qnt: Quantitative

Total protein (6.4 - 8.3 g/dL)	6.2
Albumin (calculated) (3.2 - 5.2 g/dL)	3.9
Albumin % (45 - 65%)	61
Alpha –1-Globulin (0.1 - 0.4 g/dL)	0.2
Alpha 1 % (3 - 6%)	3
Alpha-2-Globulin (0.5 - 0.9 /dL)	0.5
Alpha 2% (6 - 13%)	7
Beta Globulin (0.5 - 1.1 g/dL)	0.6
Beta Percent (11 - 19%)	10
Gamma Globulin (0.5 - 1.5 g/dL)	1.2
Gamma Globulin % (9 - 20%)	20
Total Prot. Sum (6.3 - 8.2 g/dL)	6.4
Total Prot. Sum % (98 - 102%)	101
Protein Electrophoresis, Serum	Normal fibrinogen present in sample
Kappa Free Light Chains QNT (0.37 - 1.94 mg/dL)	3.63
Lambda Free Light Chains QNT (0.57 - 2.63 mg/dL)	3.65
Free Kappa/Lambda Ratio (0.26 - 1.65)	0.99

**Figure 1:**
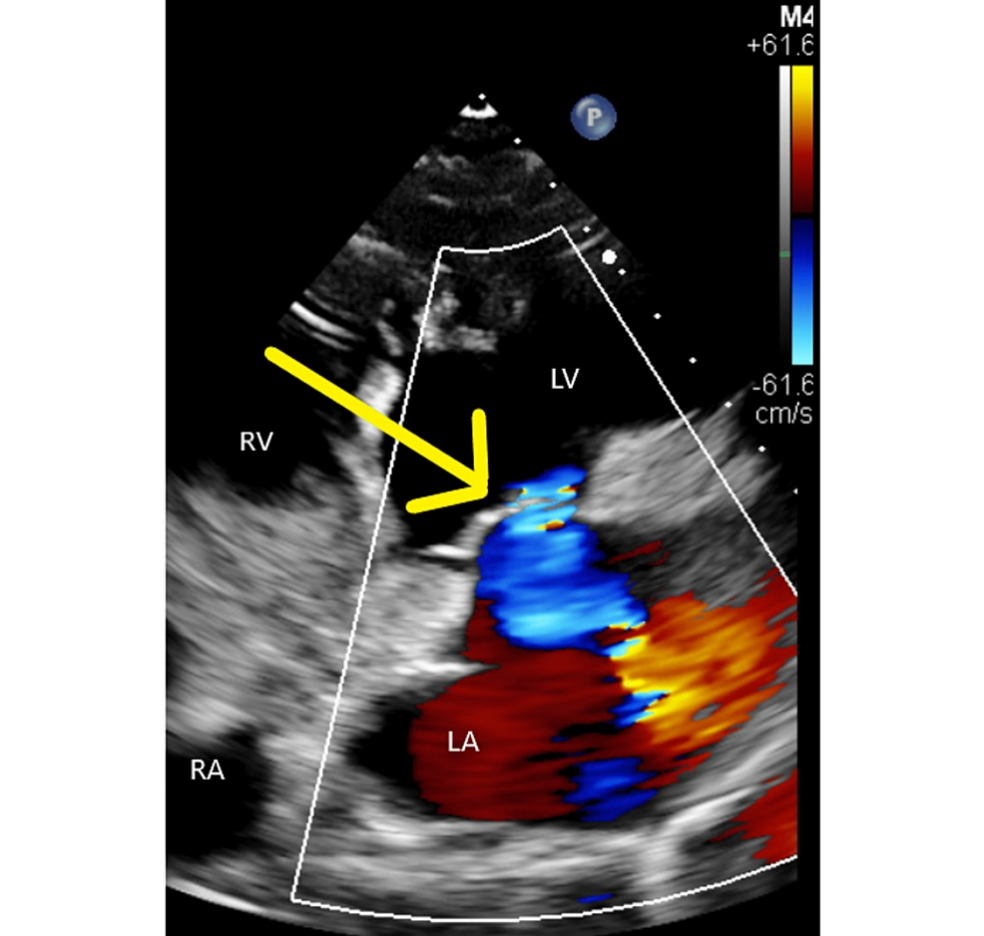
Parasternal view of transthoracic echocardiogram The yellow arrow points to the prosthetic mitral valve. LA: Left Atrium. The left atrium appears dilated with the mixed colors indicating regurgitant blood flow from the left ventricle to the left atrium through the prosthetic mitral valve. LV: Left ventricle, RV: Right ventricle, RA: Right atrium

Given the patient’s advanced age and presentation, congenital causes of hemolytic anemia were considered as low suspicion and a presumptive diagnosis of non-autoimmune hemolytic anemia was made. Due to the history of the prior mechanical mitral valve, cardiology was consulted and a transthoracic echocardiogram (TTE) was obtained to check for prosthetic mitral valve dysfunction which could be causing mechanical hemolysis (Figure 1). The TTE showed that the prosthetic mitral valve was normal in appearance and function with a mean gradient of 3 mmHg. The left atrium appeared severely dilated with mild to moderate mitral regurgitation. There was increased right ventricular systolic pressure with pulmonary hypertension. Global left ventricular systolic function was reduced with ejection fraction (EF) of 39% and leftward compression of intra-ventricular septum indicating right ventricular pressure overload.

Due to the patient’s advanced age and co-morbidities, a multidisciplinary discussion between the patient and all the teams involved in her care was held. It was decided that she was not a candidate for mitral valve extraction or replacement, therefore transesophageal echocardiogram (TEE) was not indicated. Since her hemoglobin had stabilized by this time after a total of three units of blood transfusions during hospitalization, the patient was discharged home with a plan for follow-up with her primary care physician: regular monitoring of hemoglobin level, oral iron, and folic acid replacement, and transfusion if needed. On subsequent follow-up, the patient’s hemoglobin was 10.5 g/dL and she remained clinically stable.

## Discussion

Our patient was diagnosed with non-autoimmune hemolytic anemia secondary to the mechanical mitral valve, after a complete gastroenterology workup which ruled out both upper and lower GI causes of blood loss and anemia. After a multi-disciplinary discussion, due to the patient’s co-morbidities and advanced age, it was determined that the risk outweighed the benefits of further invasive workup or management ranging from a TEE to cardiac catheterization and percutaneous or surgical valvular repair/extraction/replacement.

Hemolytic anemia can be defined as anemia that is due to premature destruction and shortened survival of RBCs, occurring either intravascular or extravascularly [5]. It can be due to numerous causes, congenital or acquired, acute or chronic, and can range from mild and asymptomatic to life-threatening severity requiring transfusions [5]. The key clue to hemolysis being the cause of anemia is a rise in reticulocyte count that cannot be explained by recent iron supplementation or bleeding [5]. Diagnosis starts with a thorough history focusing on onset and duration of symptoms, bleeding history or family history of blood disorders, history of blood transfusions, new medications, etc. [5,6]. The physical exam includes looking for pallor, icterus, splenomegaly, and cardiac murmurs. Laboratory studies will show low hemoglobin, low hematocrit and red cell count, elevated reticulocyte count, and signs of RBC destruction such as elevated LDH, indirect hyperbilirubinemia, and low haptoglobin, while peripheral smear can show schistocytes such as those seen in our patient, thereby pointing towards shearing as a cause of hemolysis [5,6]. Coombs test, flow cytometry, and further testing for enzymatic dysfunction can all help to further elucidate the primary cause. In our patient the Coombs test was negative and there was no history of familial bleeding disorders, recent infection, fever, or medication changes, so all signs pointed towards mechanical hemolysis due to mechanical valve, especially in the setting of sub-therapeutic INR.

The sudden occurrence of hemolytic anemia following a normally functioning mechanical valve as manifested by our patient after 20 years, is rare and commonly occurs in the presence of prosthetic regurgitation either through or around the prosthesis [7]. In such cases, the severity of the hemolysis depends on the level of the shear stress and not the site of regurgitation. Suggested mechanisms of hemolysis include sudden deceleration of the jet by a solid structure, or fragmentation of the jet by a solid structure, or the presence of a small anatomical regurgitant orifice [7]. While our patient did not undergo a TEE, the initial transthoracic echocardiogram (TTE) provides information on cardiac function, chambers, and valves as well as potentially identifying paravalvular leaks using the color Doppler. However, it is important to note that prosthetic valves can produce acoustic artifacts thereby masking the presence or severity of the mitral jet [6]. Further advancement in cardiac imaging has shown the increasing value of three-dimensional TEE which defines the extent of the valvular leakage more accurately in comparison to multiplane TEE [7]. Unfortunately, records could not be obtained as to why the patient required mechanical valve replacement following her initial porcine mitral valve, and the patient herself did not remember the cause.

Indications for repair either through percutaneous or surgical approach generally include the severity of hemolysis based on either persistent symptoms of fatigue, the continued requirement of blood transfusions, or else progression of valvular dysfunction [8]. However, if hemoglobin remains stable, symptoms resolve, there is minimal functional impairment, or the risk of comorbidities is greater than the benefits of invasive intervention, as seen in our patient, then an initial trial of medical therapy with iron, folate, B12 supplementation, and regular hemoglobin monitoring to correct the anemia is prudent [8].

## Conclusions

In all patients with prosthetic valve placement who present with anemia or hyperbilirubinemia, hemolysis is an important differential that must be ruled out, irrespective of the duration of a valve replacement. Our case highlights this important fact by presenting a patient who had remained asymptomatic for more than 20 years after mechanical mitral valve placement until she presented with symptoms of fatigue, dark stools, and low hemoglobin. Equally important are the indications and contraindications of invasive management of mechanical hemolysis due to prosthetic valves; our case displays a valuable example in which medical therapy was preferred over the surgical approach of valve assessment and repair.
